# Penetration rates into the stratum corneum layer: A novel quantitative indicator for assessing skin barrier function

**DOI:** 10.1111/srt.13655

**Published:** 2024-03-13

**Authors:** Suji Yoo, Jongwook Kim, Eui Taek Jeong, Seung Jin Hwang, Nae‐Gyu Kang, Jinyong Lee

**Affiliations:** ^1^ R&D Center LG H&H Co., Ltd. Seoul South Korea

**Keywords:** fluorescence microscope, skin barrier, stratum corneum, tape‐stripping, transepidermal water loss, visualization

## Abstract

**Background:**

The stratum corneum (SC), the outermost layer of the skin epidermis, acts as an effective bi‐directional barrier, preventing water loss (inside–outside barrier) and entry of foreign substances (outside–inside barrier). Although transepidermal water loss (TEWL) is a widely‐used measure of barrier function, it represents only inside‐outside protection. Therefore, we aimed to establish a non‐invasive method for quantitative evaluation of the outside–inside barrier function and visually present a skin barrier model.

**Materials and methods:**

Skin barrier damage was induced by applying a closed patch of 1% sodium dodecyl sulfate to the forearms of eight participants; they were instructed to apply a barrier cream on a designated damaged area twice daily for 5 days. The SC barrier was evaluated by measuring TEWL and fluorescein sodium salt penetration rate before, immediately after, and 5 days after damage. The penetration rate was assessed using tape‐stripping (TS) technique and fluorescence microscopy.

**Results:**

The rates of fluorescein sodium salt penetration into the lower layers of SC differed significantly based on the degree of skin barrier damage. The correlation between penetration rate and TEWL was weak after two rounds of TS and became stronger after subsequent rounds. Five days after skin barrier damage, the penetration rate of all layers differed significantly between areas with and without the barrier cream application.

**Conclusion:**

Our findings demonstrated that the penetration rate was dependent on skin barrier conditions. The penetration rate and corresponding fluorescence images are suitable quantitative indicators that can visually represent skin barrier conditions.

## 1 INTRODUCTION

The skin is crucial for protecting the body from various external factors. The stratum corneum (SC), the outermost layer of the skin, is composed of piles of dead corneocytes and an intercellular lipid matrix.^1^ This layer serves as an effective barrier, preventing excessive water loss from the body (inside–outside barrier) and invasion of harmful foreign agents such as allergens (outside–inside barrier).^2^ These two protective functions are important for human health, and a defective skin barrier may lead to skin diseases, such as atopic dermatitis, contact dermatitis, and psoriasis.^3^, ^4^, ^5^ Certain moisturizers, such as creams with lipid content, can help improve the skin's barrier function.^6^ Thus, accurate measurement of skin barrier function is important, as it can contribute to checking and maintaining skin health.

Transepidermal water loss (TEWL) is a widely‐used general method of measuring skin barrier function^7^; numerous studies involving atopy and psoriasis have used TEWL for this purpose.^8^ Because TEWL is an indicator of the inside–outside barrier, several studies have attempted to assess the outside–inside barrier function using the transdermal penetration method. Lotte et al.^9^ reported a linear correlation between percutaneous absorption of radiolabeled compounds and TEWL at four anatomical sites in men. Lavrijsen et al.^10^ found a significant correlation between TEWL and vascular response to hexyl nicotinate penetration in patients with keratinization disorders. While these studies successfully quantified the outside–inside barrier function, they did not visually demonstrate the function. Katsuta et al.^11^ used fluorescein and fluorescence microscopy to visually demonstrate the outside–inside skin barrier function of a sensitive skin and reported a correlation between the intensity of penetrated fluorescein and TEWL.

The tape‐stripping technique non‐invasively removes corneocytes layer‐by‐layer from the SC,^12^ and it is often used in research related to skin penetration. Tape‐stripping techniques have been used to quantify the penetration of drugs into the skin^13^ and to study skin penetration enhancers.^14^ Fluorescein sodium salt is a representative fluorescent substance commonly used in ophthalmology,^15^ as it is harmless to the human body.^16^ Furthermore, it has been used in studies on skin health, such as studies on the correlation between liposome fluidity and transdermal drug delivery^17^ and those on the protective effect of barrier creams on the skin surface.^18^ In a solution, fluorescein sodium salt absorbs blue light and emits green to yellow fluorescence.

In this study, we aimed to establish a non‐invasive method to quantitatively evaluate outside–inside skin barrier function. TEWL, a representative indicator of barrier function, is a numerical value that allows absolute comparison of barrier function but does not depict the skin barrier model visually. Therefore, in this study, we used the tape‐stripping technique and fluorescein sodium salt to visualize skin barrier function in an SC penetration model.

## 2 MATERIALS AND METHODS

### 2.1 Clinical study

The study was conducted in accordance with the ethical principles of the Declaration of Helsinki and was approved by our Institutional Review Board (LGHH‐20230511‐AA‐05‐01). All participants received an explanation of the purpose of the study and provided written informed consent before participation.

Eight healthy, Korean volunteers (aged 28−35 years) were enrolled in this study. People with skin diseases such as eczema, atopy, skin allergies, or hypersensitivity and those who were pregnant or lactating were excluded from the study. The participants were instructed not to use any leave‐on products on their forearms the day before or on the morning of the study. Disruption of skin barrier function was induced by applying a 1% sodium dodecyl sulfate (SDS) solution to the test areas of 1 × 1 cm^2^ on the forearm for 24 h; the participants were instructed to use a barrier cream on a designated damaged area twice a day for 5 days. The area where each participant was to apply a barrier cream was randomly assigned.

Before skin barrier damage (normal), immediately after damage (damaged), and 5 days after damage (partially recovered), TEWL was measured and fluorescence images of the skin were collected for quantitative assessment of skin barrier function. On day 5 after damage, the skin areas where the barrier cream was applied and not applied were measured separately to determine the differences in skin barrier function. The participants cleaned their forearms before the measurements and adjusted to an air‐conditioned evaluation room for 20 min. The temperature was 22 ± 2°C and relative humidity was 50 ± 10%.

TEWL was measured using Tewameter TM Hex (C+K electronic GmbH, Köln, Germany). TM Hex measures water evaporation from the skin. Subsequently, a 0.1% solution of fluorescein sodium salt was applied occlusively for 10 min to the same area on the forearm, and images of the skin surface were taken using a fluorescence microscope. To observe the degree of penetration of the reagent into the SC, it was stripped five times, layer‐by‐layer, using a tape‐stripping procedure using D‐SQUAME tape (Clinical and Derm, Dallas, TX, USA), with fluorescence microscopy performed after each procedure.

### 2.2 Fluorescence microscopy

Fluorescein sodium salt was obtained from Sigma Aldrich (St. Louis, Missouri, USA). The reagent was diluted to 0.1% with distilled water.

**FIGURE 1:**
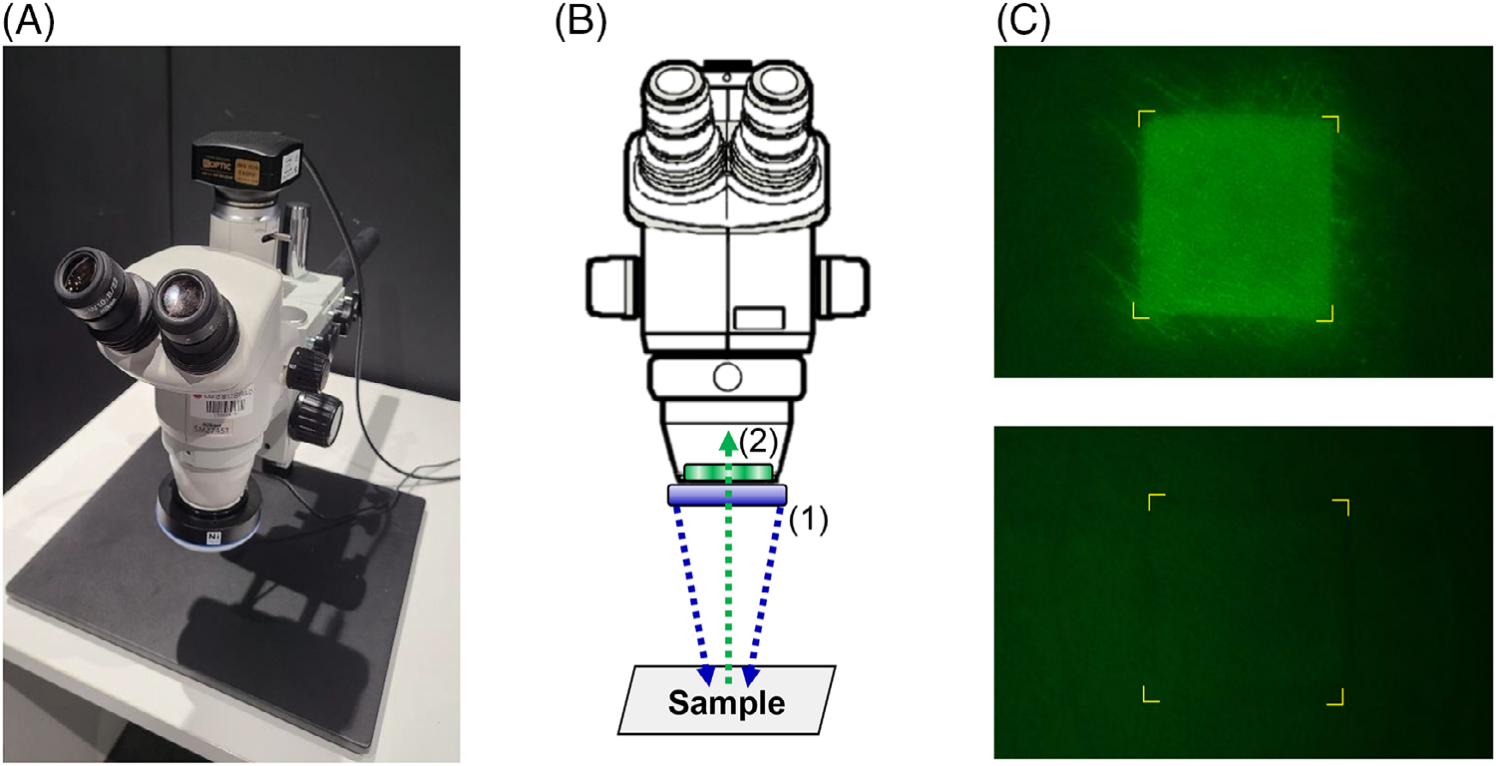
(A) Photograph of a manufactured microscope designed for observing fluorescent sodium reagent. (B) Principle of observing a fluorescent sample on the skin using the microscope. Blue light from a ring‐type LED light source excites the sample (1); green light with a wavelength of 520−530 nm, emitted from the sample, passes through the bandpass filter (2), and is observed by the camera. (C) Comparison of images of the skin, with specific substances applied, observed under the microscope (upper: fluorescein sodium salt; lower: distilled water). LED, light emitting diode.

**FIGURE 2:**
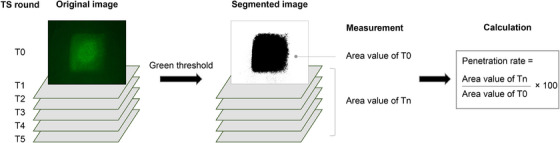
Sequential explanation of fluorescence image analysis and the penetration rate calculation method.

For direct, non‐invasive measurement of the amount of fluorescent reagent penetrating the skin, a fluorescence microscope was first manufactured (Figure 1a). A ring‐type blue light emitting diode (LED) light source and green wavelength bandpass filter were mounted on the body of a stereoscopic zoom microscope SMZ745T (NIKON, Tokyo, Japan) to measure the fluorescein sodium salt. Figure 1b illustrates the measurement principle. Fluorescein sodium salt on the skin surface absorbs blue light from the light source and emits wavelengths of 520−530 nm through the lens after passing through the bandpass filter. Fluorescence microscopy results were verified by comparing the fluorescence images of the skin with 0.1% solution of fluorescein sodium salt to those with water application (Figure 2).

### 2.3 Fluorescence image analysis

ImageJ software was used to quantitatively determine the area of fluorescent regions in the image. Six areas were measured using the same green (G) threshold from the fluorescence images obtained for each round of tape‐stripping (T0–T5) on a single skin area. Each round was labeled as Tn, where n corresponded to the number of tape‐stripping. To evaluate the degree of penetration of fluorescein sodium salt into the skin compared to that at the skin surface, the penetration rate for each SC layer was calculated as the ratio of the area of Tn to that of T0 multiplied by 100. The overall fluorescence image analysis is illustrated in Figure 2.

Additionally, a three‐dimensional (3D) structure‐like skin model was created through graphic design using fluorescent images to visualize the penetration of foreign substances. Color conversion of the original images was performed using ImageJ software. Subsequently, Cinema 4D and Photoshop CS6 software were used to stack the images vertically and apply a gradient that gradually blurred the images from top to bottom.

### 2.4 Statistical analysis

Data were analyzed using Jamovi 2.2.5 (The Jamovi project, 2019). Statistical comparisons were conducted using the Friedman test with post‐hoc analysis (Conover's test with Bonferroni correction) among the three groups with different degrees of skin barrier damage, namely normal, damaged, and partially recovered. The Wilcoxon signed‐rank test was used for comparisons between the groups with and without barrier cream application. The correlations between TEWL and penetration rate were analyzed by calculating the Pearson correlation coefficients (r). *p‐*values < 0.05 were considered statistically significant in all analyses.

## 3 RESULTS

### 3.1 Comparison of penetration rates according to skin barrier conditions

**FIGURE 3:**
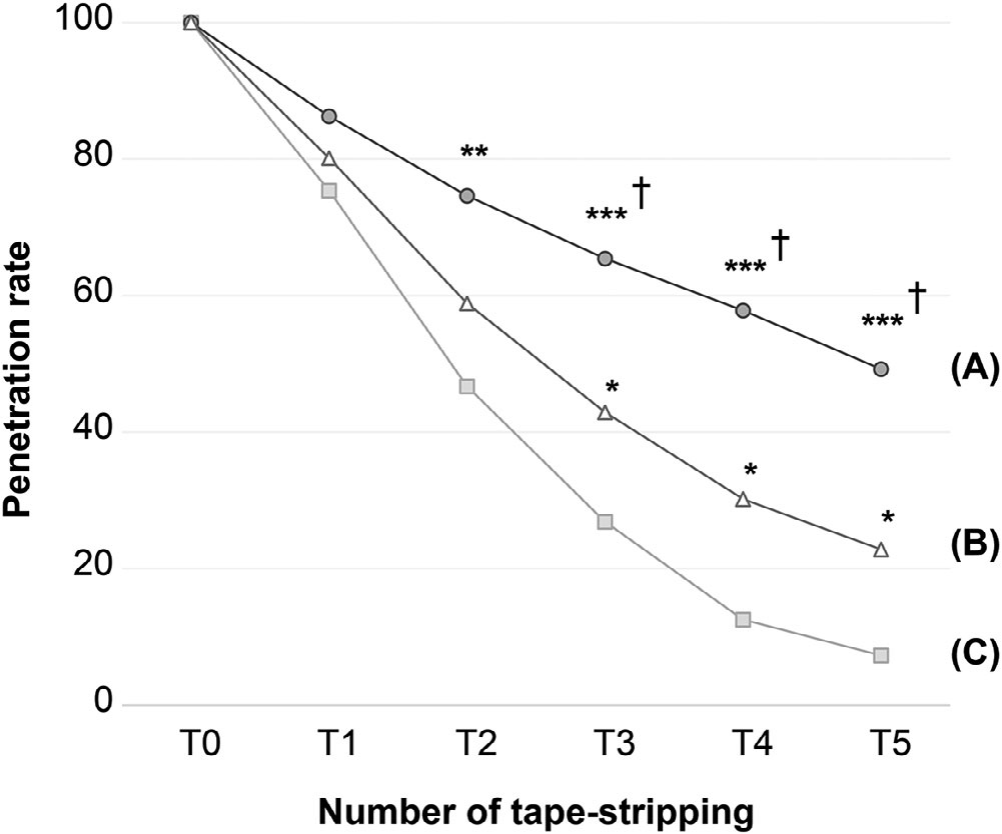
Comparison of the changes in the penetration rate of fluorescein sodium salt according to the number of tape‐stripping procedures in the three groups, that is, (A) skin immediately after damage by SDS, (B) skin 5 days after damage, and (C) normal skin †*p* < 0.05, significantly different compared to (B) *p < 0.05, **p < 0.01, ***p < 0.001, significantly different compared to (C) SDS, sodium dodecyl sulfate.

**FIGURE 4:**
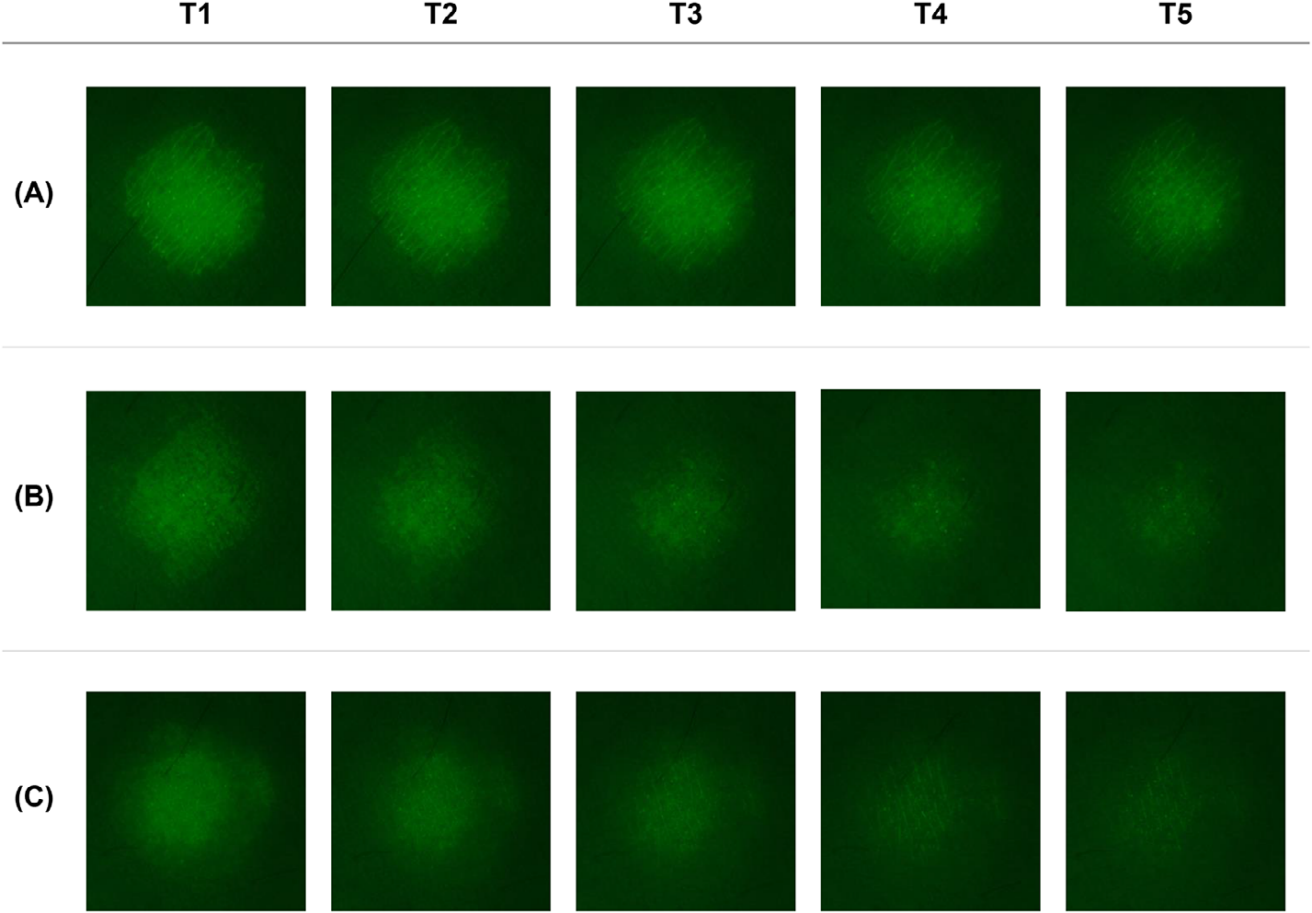
Representative images of fluorescein sodium salt penetrating the skin, after tape‐stripping (T1–T5) in each group, as observed under a microscope: (A) skin immediately after damage by SDS, (B) skin 5 days after damage, and (C) normal skin SDS, sodium dodecyl sulfate.

We quantitatively analyzed the skin fluorescence images following repetitive tape‐stripping procedures before skin barrier damage, immediately after damage, and 5 days after damage to compare the penetration rates of the fluorescein sodium salt into the SC under different skin barrier conditions. The penetration rate, in all groups, gradually decreased from the skin surface to the deeper layers. The overall tendency of the penetration rate from T1 to T5 was found to be higher in the following order: damaged, partially recovered, and normal skin, indicating that the penetration rate of the reagent increased as the skin barrier function decreased (Figure 3). The gap in penetration rates between the groups became more evident in the deeper layers of the skin. There was a significant difference in penetration rates between the normal and damaged groups at T2 (*p* < 0.01). In addition, from T3 to T5, significant differences were observed among all pairwise comparisons of the three groups (*p* < 0.001 between normal and damaged, *p* < 0.05 between normal and partially recovered, and *p* < 0.05 between damaged and partially recovered). Figure 4 shows the representative fluorescence images of each skin layer after repetitive tape‐stripping in each group.

### 3.2 Correlation between penetration rate and TEWL

**FIGURE 5:**
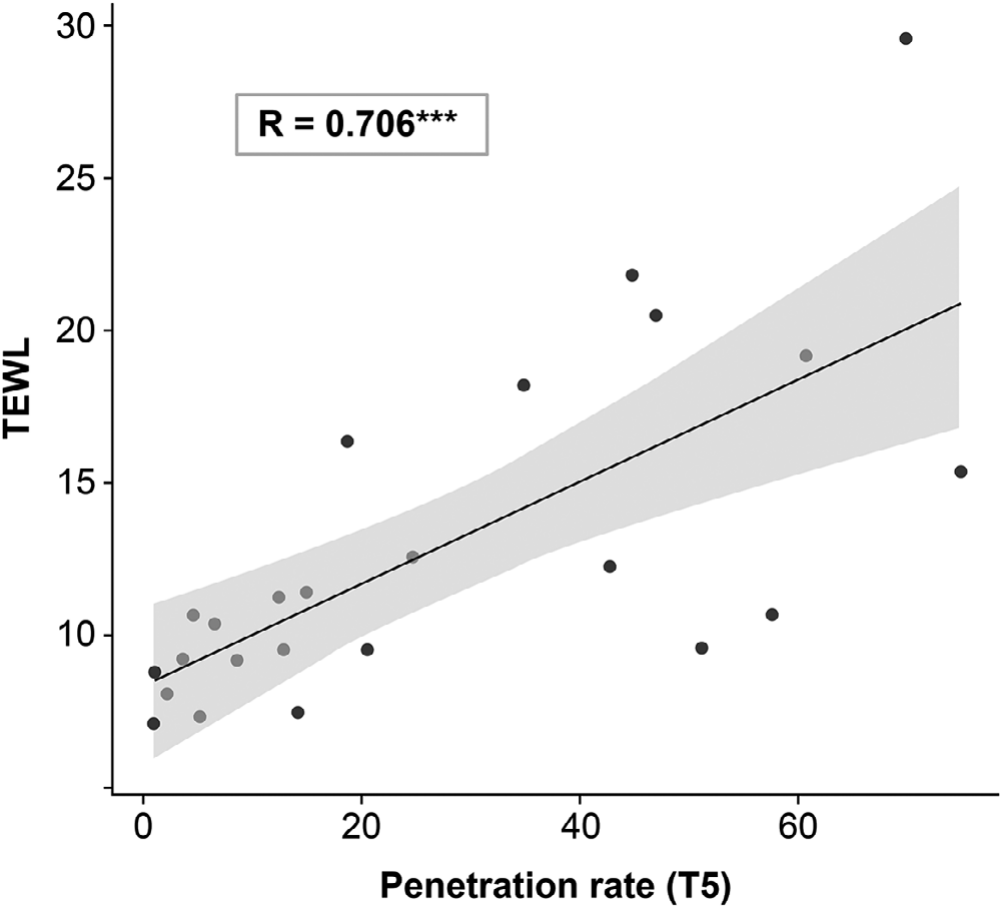
Scatterplot of values of the relationship between penetration rate (T5) and TEWL The correlation was analyzed using Pearson's correlation test; R indicates the correlation coefficients (***p < 0.001) TEWL, transepidermal water loss.

Table 1 lists the correlations between the penetration rate and TEWL. No correlation was observed during the first round of tape‐stripping (T1). However, a weak correlation (*R* = 0.486, *p *< 0.05) was observed for T2 and, gradually, stronger correlations were observed for T3 (*R* = 0.547, *p *< 0.01), T4 (*R* = 0.648, *p *< 0.001), and T5 (*R* = 0.706, *p *< 0.001). Distribution of the values for the relationship between T5 penetration rates and TEWL is shown in Figure 5.

**TABLE 1:** Correlation coefficients between the penetration rate and transepidermal water loss (TEWL).

	Penetration rate				
Round of tape‐stripping	T1	T2	T3	T4	T5
TEWL	0.258	0.486*	0.547**	0.648***	0.706***

*Note*: Values of *p < 0.05, **p < 0.01, and ***p < 0.001 were determined using Pearson's correlation test.

### 3.3 Comparison of penetration rates based on barrier cream application

**FIGURE 6:**
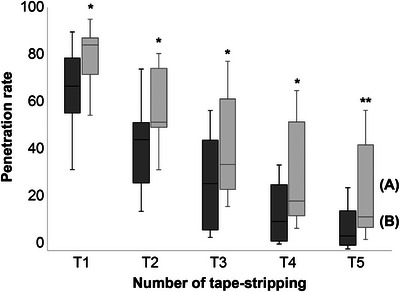
Comparison of penetration rates of fluorescein sodium salt using the number of tape‐stripping rounds between the areas without (A) and with (B) barrier cream application *p < 0.05, **p < 0.01, significant difference between (A) and (B).

**FIGURE 7:**
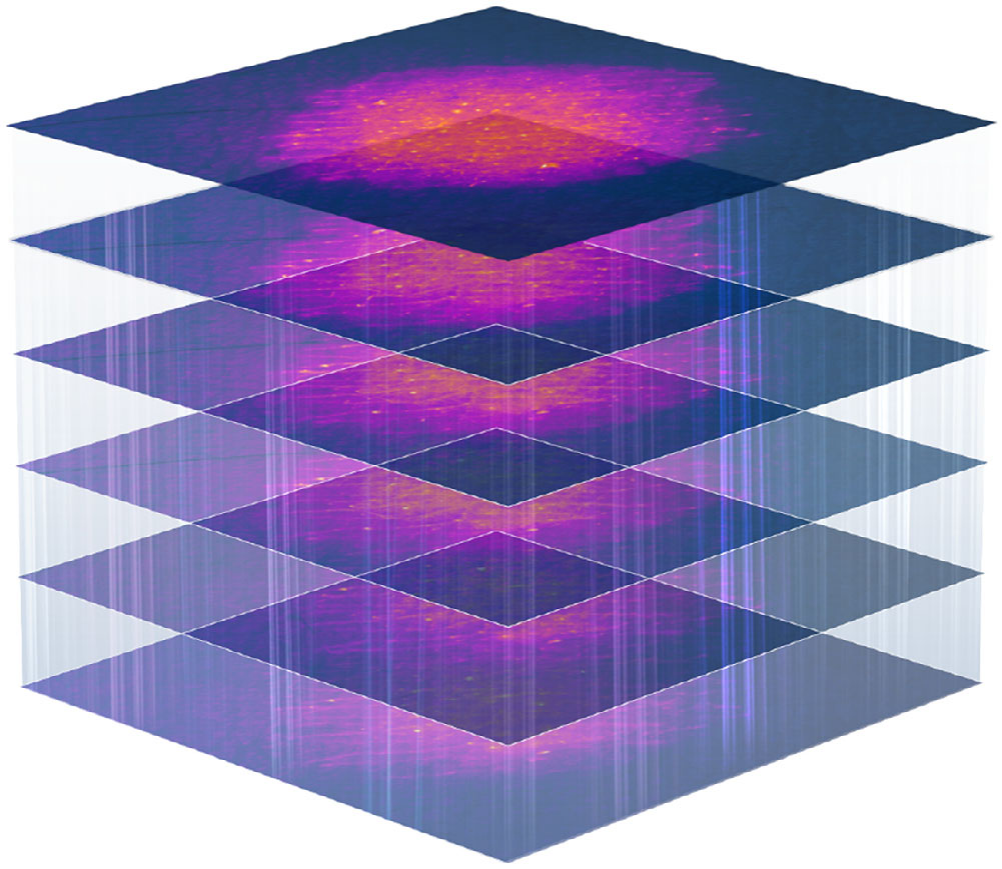
Visualization of skin barrier image, illustrating the extent of fluorescein sodium salt penetration into each layer.

To investigate the differences in recovery of skin barrier function, depending on barrier cream application, fluorescence images of skin areas with and without the cream application taken 5 days after damage were analyzed. Penetration rates in the areas with the cream applied from T1 to T5 were 65.88, 42.94, 27.93, 15.49, and 8.84, respectively; the values were relatively lower in all layers compared to those in areas without cream applied (80.14, 58.86, 42.88, 30.17, and 22.81, respectively) (Figure 6). There was a significant difference in penetration rates between the two areas in all layers. The results collectively indicate that restoration of the skin barrier damage, through barrier cream application, can be objectively assessed by comparing the differences in the penetration rates of fluorescent reagents. A 3D structure‐like skin model demonstrating the penetration of foreign substances is shown in Figure 7.

## 4 DISCUSSION

With increasing interest in skin health since the COVID‐19 pandemic, improving the function of the skin barrier has become a major goal in skincare. However, few studies on objective methods of evaluating skin conditions have been published. In the present study, we demonstrated that the penetration rate of foreign substances into the skin, investigated using non‐invasive in vivo experiments, can represent skin barrier status.

Our results showed that the penetration rate was dependent on the extent of skin barrier damage; the damaged skin barrier exhibited a significantly higher penetration rate than did the undamaged barrier. Furthermore, as the skin was partially repaired, the penetration rate became lower. We used 1% SDS solution to induce surfactant‐induced damage to the skin barrier. Several studies have previously reported the potential of SDS to disrupt the integrity of SC. Topical application of SDS causes disturbance and transformation of epidermal lipids, which are known to morphologically represent the permeability barrier.^19^, ^20^ Moreover, Lips et al.^21^ demonstrated that anionic surfactants, such as SDS, enter the SC and cause protein denaturation. As a consequence of these interactions, the damaged skin barrier cracks open between the corneocytes^22^ and loses the ability to control penetration of foreign substances. This could explain our results, where a relatively higher penetration rate of fluorescein sodium salt was observed in more severely damaged skin barriers.

Penetration rates were calculated from fluorescence images for each round of tape‐stripping, for up to five rounds. While Yoshida et al.^23^ reported a relationship between fluorescent staining and skin surface conditions with tape‐stripping methods, we focused on outside‐inside barrier function by assessing the penetration rates of the fluorescent reagent. We considered that penetration to a depth of approximately 2 µm was observed because approximately 0.4 µm of the SC is removed with each strip.^24^ We set the maximum number of tape‐stripping to five as a standard, beyond which no further penetration would be observed in a normal skin. However, the fluorescent reagent was observed in the damaged skin even after five rounds of tape‐stripping, implying that there is a high possibility of penetrating substances remaining on the damaged skin even after further tape‐stripping. A limitation of this study is that we did not proceed with additional tape‐stripping; therefore, we could not confirm the actual depth of penetration in the damaged skin.

We established a strong correlation between the penetration rate and TEWL. Both parameters represent the permeability of skin barrier but in opposite directions. TEWL, which indicates the water vapor lost across the skin from the body,^25^ represents in–out permeability, whereas the penetration rate, which indicates the quantitative proportion of substance that has entered the skin from the external environment, represents out–in permeability. As mentioned previously, TEWL is the most widely‐used parameter for assessing skin barrier functions in numerous studies.^7^, ^8^ To validate the suitability of penetration rate as another objective indicator of skin barrier, we conducted a correlation analysis between the two parameters. Based on our findings, similar to TEWL, penetration rate can be useful for evaluating the health of the skin barrier.

Moreover, we found that application of barrier cream resulted in a lower penetration rate than that in an untreated skin. Certain moisturizers enhance skin barrier function; low‐molecular‐weight humectants in creams, such as glycerin, keep the SC well hydrated by attracting water.^26^, ^27^ Moisturizers with lipid content help to accelerate the restoration of the skin barrier function after 5 days of treatment.^28^ The lipids may prevent water escape from the skin by forming a film.^29^ Further, they may penetrate the skin and affect the barrier properties.^30^, ^31^ Their entry into the lipid layers surrounding the corneocytes could maintain the moisture levels and prevent crack formation in the SC.^32^ Thus, cream application is believed to improve skin barrier density weakened by damage and restore its defensive function against foreign substances. This result indicates that the penetration rate can be used as an indicator of the skin repair efficacy of skin care products. In addition, utilizing layer‐specific fluorescence images, it can serve as a tool to visualize the improved protective function of the skin barrier after applying skin care products. Future studies are being planned to evaluate multiple products with various formulations.

## 5 CONCLUSIONS

Our study demonstrated that the penetration rate of foreign substances differs depending on the degree of skin barrier damage. Similar to TEWL, the penetration rate is useful in quantitatively assessing skin barrier properties. Additionally, a 3D skin model image, generated using layered fluorescence images, can be used as an effective indicator of the health status of the skin barrier.

## AUTHOR CONTRIBUTIONS

Suji Yoo, Jongwook Kim, Jinyong Lee, and Eui Taek Jeong designed the research study. Suji Yoo and Jongwook Kim performed the research, analyzed the data, and wrote the paper. Jinyong Lee, Seung Jin Hwang, and Nae‐Gyu Kang contributed to supervision and project administration. All authors have read and approved the final manuscript.

## CONFLICT OF INTEREST STATEMENT

The author declares no conflicts of interest.

## ETHICS STATEMENT

The study was conducted in accordance with the ethical principles of the Declaration of Helsinki and was approved by our Institutional Review Board.

## Data Availability

The data that support the findings of this study are available from the corresponding author upon reasonable request.
